# The psychosocial experiences of human papillomavirus (HPV) positive oropharyngeal cancer patients following (chemo)radiotherapy: A systematic review and meta‐ethnography

**DOI:** 10.1002/pon.5984

**Published:** 2022-07-05

**Authors:** Sara Matthews, Jo Brett, Pras Ramluggun, Eila Watson

**Affiliations:** ^1^ Oxford Institute of Nursing Midwifery and Allied Health Research (OxINMAHR) Faculty of Health and Life Sciences Oxford Brookes University Oxford UK

**Keywords:** cancer, head and neck cancer, human papillomavirus, meta‐ethnography, oncology, oropharyngeal cancer, Psycho‐Oncology, psychosocial, qualitative, radiotherapy

## Abstract

**Objective:**

The UK incidence of oropharyngeal cancer has risen sharply over the last 30 years with an increase in human papillomavirus (HPV) associated diagnoses, most prevalent in younger, working age populations. This meta‐ethnography explores the psychosocial needs of HPV+ve oropharyngeal cancer patients during early recovery following (chemo)radiotherapy.

**Methods:**

Meta‐ethnography methods were used, based on the approach of Noblit and Hare. Systematic searches for relevant qualitative studies were conducted in five electronic databases (MEDLINE, PubMed, CINAHL, PsycINFO and Cochrane database) between 2010 and 2021, followed by citation searching.

**Results:**

Twenty‐three papers exploring the psychosocial needs of HPV+ve oropharyngeal cancer patients after treatment were included. Findings were synthesised to develop five constructs: ‘gaps in continuity of support from healthcare professionals’ reflecting unmet needs; ‘changes to self‐identity’ revealing the comprehensive disruption of this disease and treatment; ‘unrealistic expectations of recovery’ highlighting the difficulty of preparing for the impact of treatment; ‘finding ways to cope’ describing the distinct complexity of this experience; and ‘adjusting to life after the end of treatment’ exploring how coping strategies helped patients to regain control of their lives.

**Conclusions:**

Completing (chemo)radiotherapy signalled a transition from hospital‐based care to home‐based support, challenging patients to address the constructs identified. An unexpectedly difficult and complex recovery meant that despite a favourable prognosis, poor psychosocial well‐being may threaten a successful outcome. The provision of tailored support is essential to facilitate positive adjustment.

## 1 BACKGROUND

Head and neck cancers (HNC) are the 6th most common cancer type globally. The UK incidence of oropharyngeal cancer quadrupled between 1990 and 2010^1^ and continues to rise^2^ with up to 80% now linked to human papillomavirus (HPV).^3^, ^4^ Disease onset in this sub‐group is typically during middle age (40–55 years)^5^ and younger than that previously seen in HNC (65 years plus) traditionally associated with tobacco and alcohol consumption. Although HPV+ve oropharyngeal disease is often locally advanced when diagnosed, it is responsive to radiotherapy, often given concurrently with chemotherapy, that is, chemoradiotherapy, (75%–80% surviving 5 years).^6^ However, poor quality of life due to severe treatment side effects is common for example, excessively dry mouth and difficult or painful swallowing,^7^ resulting in significant post‐treatment support needs.

Over the last decade, qualitative research has explored HNC patients' experiences, resulting in four pertinent reviews. The first^8^ revealed disruption in all aspects of life, diminishing a sense of self, managed by finding support, re‐evaluating what was important and adapting to the future. Mixed HNC populations included those who had surgery, with different experiences to those primarily receiving (chemo)radiotherapy (e.g., facial disfigurement, oral reconstruction, and prosthetics). A review of the psychosocial impact of HPV+ve HNC diagnosis^9^ found quality of life lowest after 2‐3 months, commonly when radiotherapy is scheduled,^1^ but included only one qualitative study,^10^ revealing a sense of stigma and negative impact upon relationships, 1–5 years later.

Two recent reviews selected (chemo)radiotherapy studies but again included mixed HNC populations and different time points along the illness trajectory, from diagnosis to long‐term survival. A meta‐ethnography of 8 studies exploring HNC radiotherapy experiences,^11^ described unmet needs related to isolation, making sense of the experience, disrupted life, waiting and uncertainty. Although some needs were met during treatment, the importance of therapeutic radiographers in building relationships with patients was emphasised to aid coping and understanding. A review of 13 studies sought to understand the impact of the lived experience of treatment upon patients.^12^ Although incorporating evaluation of both early and late recovery phases, combining differing temporal perspectives and ‘response shifts’,^13^ areas for psychosocial research were identified, including approaches to address feelings of post‐treatment abandonment.

Reviews of mixed HNC populations leave gaps in understanding about the distinct psychosocial experiences and support needs of the growing population of HPV+ve oropharyngeal cancer patients following (chemo)radiotherapy. Management guidelines for this population in the UK following the PETNeck trial^14^ mean patients wait for a 12‐week post‐treatment response assessment scan to determine if a neck dissection is required.^15^ This time of waiting encompasses a transition from hospital‐based support for management of side effects as they peak, to home‐based self‐care with hospital follow up. Early research of this *‘hidden experience’*,^16^ when patients need a *‘hand to hold’* ^17^ alongside increased understanding of treatment sequelae,^18^ has contributed to developments in MDT support including pre‐ and post‐habilitation for example, physical exercise, nutrition and swallowing.^19^, ^20^ Contemporaneous adoption of Intensity Modulated Radiotherapy (IMRT), a targeted form of treatment, has also changed patients' experiences.^21^ It is therefore timely for this review of patients' experiences during early recovery, addressing the question: ‘What are the psychosocial experiences and needs of HPV+ve oropharyngeal cancer patients following (chemo)radiotherapy?’

## 2 METHOD

Meta‐ethnography methods, as set out by Noblit and Hare,^22^ were used to synthesise existing qualitative research. New insight was developed through translation of one study into that of others in order to develop a ‘line of argument’ through reciprocal, or potentially refutational interpretation.^23^

### 2.1 Search strategy

The search strategy was developed using Population, Exposure, Outcome (PEO)^24^ and keywords identified in preliminary searches. The resultant free‐text, Medical Subject Headings (MeSH) and thesaurus terms were used in searches adapted for MEDLINE, PubMed, CINAHL, PsycINFO and Cochrane databases. Multiple psychosocial terms related to the emotional, psychological, and social consequences of HPV+ve oropharyngeal cancer were included to ensure a sensitive search (Table S1). Citation searching included pertinent papers alongside the ‘Web of Science’ database.

### 2.2 Eligibility criteria

Criteria were developed through discussion between the authors (Table 1).

**TABLE 1:** Eligibility criteria

Inclusion criteria	Exclusion criteria
Qualitative HNC studies with analysis of psychosocial experience or support needs of patients during early recovery following (chemo)radiotherapy	Quantitative or mixed methodologies focused on physical or functional QoL without analysis of psychosocial needs
	Explorations of patient experience only at diagnosis, during (chemo)radiotherapy or long‐term survivorship
(Chemo)radiotherapy HNC studies with at least 1/3^rd^ oropharyngeal patients, or if site not given, at least 1/3^rd^ study's patients aged 40–65 (typical of oropharyngeal patients)^a^	Studies including mixed cancers or only surgical or palliative patients
Primary studies, peer reviewed	Healthcare professionals' experiences only
Published: Jan 2010–Sep 2021, reflecting ongoing rising incidence of HPV+ve oropharyngeal cancer and widespread adoption of IMRT	Evaluations of different (chemo)radiotherapy treatments or of rehabilitation or self‐management interventions
English language	Expert opinion papers or conference abstracts
Patients 18 years and above	

^a^ For studies including surgical patients but not specifying HNC site, the sample was assumed to reflect the UK HNC population that is, 25% oropharyngeal cancers^4^.

### 2.3 Selection of studies

Titles and abstracts were screened for eligibility by SM, with a random 5% sample of excluded papers confirmed by the co‐authors.

### 2.4 Quality appraisal

Eligible studies were appraised using the Critical Appraisal Skills Programme (CASP) qualitative checklist^25^ to assess reliability for inclusion. This commonly used checklist enabled structured assessment of 10 items of study quality and insight into content.

### 2.5 Data extraction, synthesis, and translation

A ‘Synthesis table’ listed the selected papers chronologically and by research foci, demonstrating knowledge and practice development with the authors' 2nd order constructs entered alongside representative quotations (1st order constructs) from which they were derived (Table S2). Included quotations were those attributable to patients under 65 years (more likely to be HPV+ve), whilst those related to experiences during treatment or surgery were disregarded, as were caregivers' quotations. SM re‐read the papers enabling immersion in their meaning and considered quotations in terms of psychosocial experiences and consequent needs. The resultant interpretations or ‘metaphors’ were entered into the table, ensuring transparency within this inherently subjective process.^22^ Following debate between authors, terminology was agreed, underpinned by theoretical knowledge. Metaphors developed were organised into the table's columns and the relationships and commonality between them considered iteratively, resulting in the evolution of ‘3rd order constructs’ which appeared later as the headings of 5 columns. Sufficient similarity was found between the studies for reciprocal translation and the development of a line of argument describing the relationships between the constructs.^22^ Any refutations were described.

## 3 RESULTS

### 3.1 Search results

**FIGURE 1:**
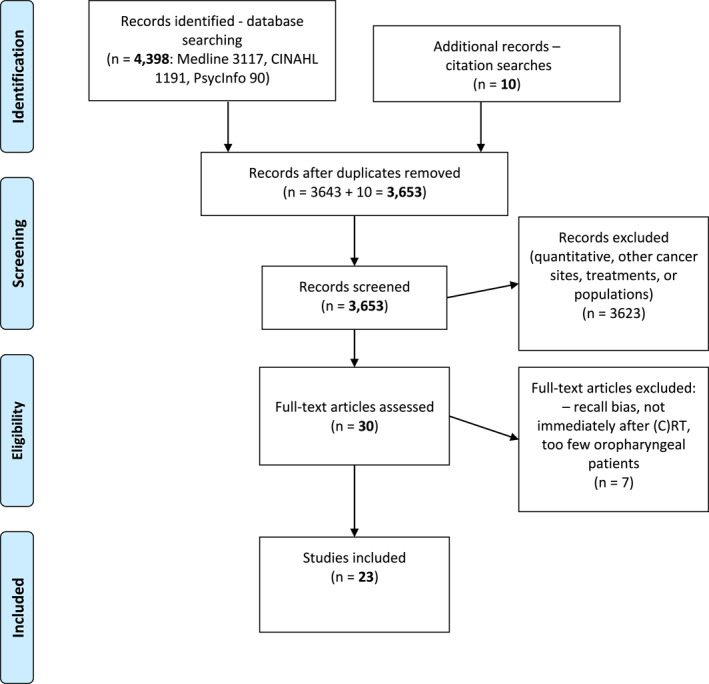
Prisma flow diagram

Following the retrieval of 4398 papers, duplicates were removed leaving 3643, with 10 added from citation searching (Figure 1). SM used the eligibility criteria to screen titles and abstracts, selecting 30 papers for full‐text review. A further 7 were ineligible following discussion with the co‐authors. 23 papers were therefore chosen, including two papers based on the same study population, but with different foci.^26^, ^27^ Quality appraisal found acceptable methodological rigor: authors set out research aims, design and findings well, ethical issues were considered but reflexivity was often not explained. Maximal purposive sampling ensured diversity within some studies,^26^, ^27^, ^28^ whilst others chose convenience sampling^29^, ^30^, ^31^ to address research questions.

### 3.2 Study characteristics

The research foci of the 23 papers fell into two groups: (Table S3).

1. Experience and psychosocial support needs (n.11), three of which addressed typical features of HPV+ve oropharyngeal cancer that is, HNC in middle adulthood, the impact of HPV and HNC as parents of young children.^32^, ^33^, ^34^
2. Experience of physical effects following radiotherapy and psychosocial impact (n.12), with 9 related to nutritional consequences (e.g., dysphagia, enteral feeding, xerostomia).

Most studies reported qualitative data at one time point (n.19) and were descriptive (n.20) using various methods for example, ‘Interpretative description’ (n.4),^35^ enabling deeper understanding built upon existing knowledge. Other research designs were Interpretative Phenomenological Analysis of the lived experience of xerostomia,^36^ ethnographic observation of eating behaviour^37^ and Grounded Theory development of a model of adjustment.^38^ Differing epistemological positions necessitated careful interpretation.^39^ Commonly, semi‐structured interviews were analysed thematically.

### 3.3 Results of synthesis

**FIGURE 2:**
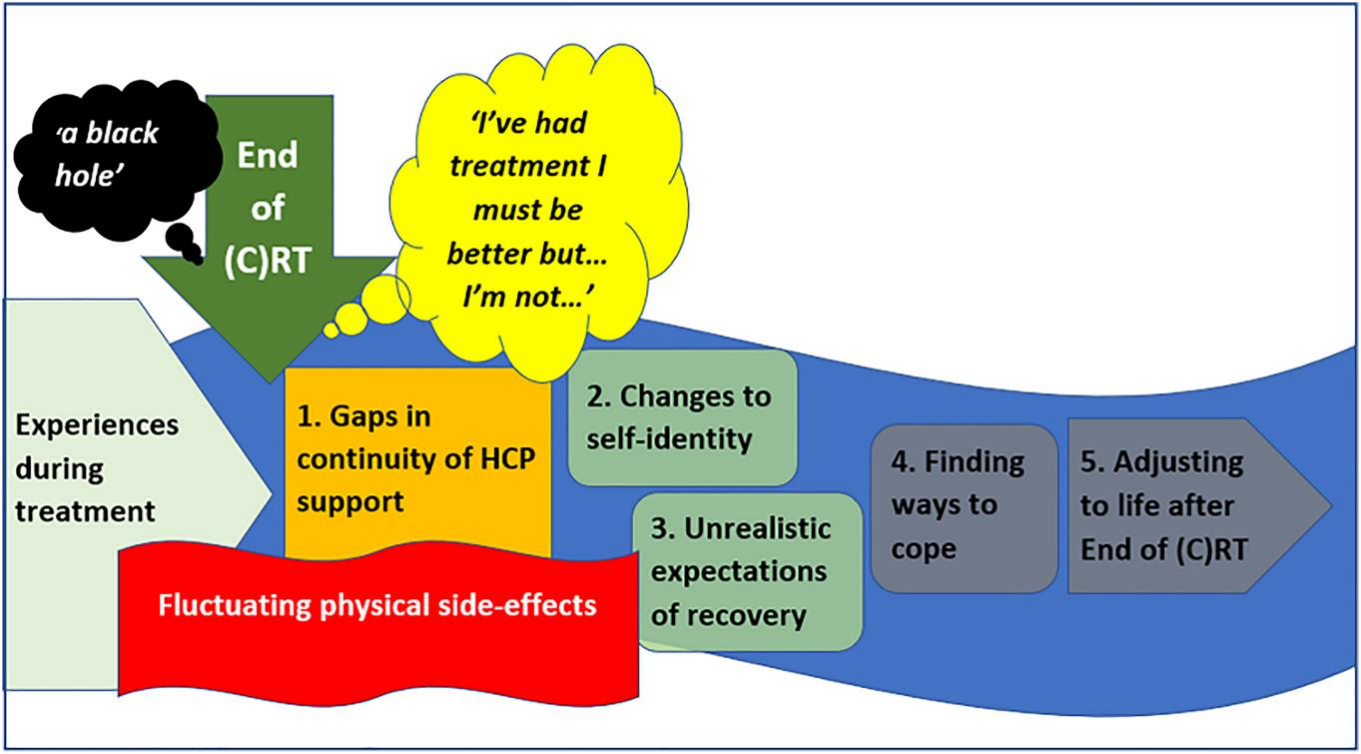
A conceptual model

Five interrelated 3^rd^ order constructs were derived: ‘gaps in continuity of support from healthcare professionals’, ‘changes to self‐identity’, ‘unrealistic expectations of recovery’, ‘finding ways to cope’, and ‘adjusting to life after treatment’ and are depicted in Figure 2.

### 3.4 Gaps in continuity of support from healthcare professionals (HCPs)

This construct revealed gaps in emotional support and information needs. Completing treatment presented patients with an illness milestone, focussing thoughts upon recovery whilst processing their treatment experiences. However, the loss of daily contact with HCPs meant fewer opportunities for support or to ask time‐sensitive questions about fluctuating side effects. Although it marked a release from intense, hospital‐imposed schedules it also brought feelings of abandonment^27^, ^40^, ^41^ and a ‘void’ of emotional support^31^ as the umbrella of care seemed removed:

When the radiotherapy started… I received support from many staff [members]…after the final radiation… then everyone disappeared.^40^

Despite patients knowing they could contact HCPs some chose to be stoic which, alongside uncertainty about when to make contact, created a barrier:

As the nurse practitioner says: “If something is wrong, just give a call.” But you don’t want to do that too soon. Still a kind of threshold, I think. You first wait for better times, just one more week.^41^

On‐going enteral feeding enabled continuity in support from trusted HCPs in recent studies.^41^, ^42^, ^43^ Unmet needs following a lack of empathy, poor communication and insufficient symptom management advice were reported in older studies where patients had *‘to learn through the school of hard knocks’*.^31^ In the absence of HCPs to ask pertinent questions to assuage concerns about the origins and disease course of HPV,^33^ patients sought written information, but found it was inapplicable:

… either aimed at preventing young people from contracting HPV or preventing older people from getting cancer through HPV.^33^

Younger patients who were parents wanted to know how to support children^34^ and HNC information seemed aimed at older patients. Where access to support for specific and timely information was perceived as inadequate, patients proposed strategies including more frequent follow ups.^40^, ^41^

### 3.5 Changes to self‐identity

This concept characterised how experiences challenged and diminished self‐identity, as lives were profoundly disrupted. Roles as parents and partners, and as providers for others were affected:

…there were some dark days where, …I didn’t think about anybody ‐ I didn’t think about my kids, I didn’t think about my wife. I thought about myself and how I had to get through this . . . And that was hard for her to hear.^32^

Disruption to daily living included pain,^36^, ^40^ lack of sleep^29^, ^31^, ^32^, ^36^, ^42^, ^44^, ^45^ and considerable time required for self‐care to manage symptoms,^42^, ^43^, ^46^ likening it to a full‐time job.

Social identity was affected by functional changes in speaking, eating, and drinking, causing social embarrassment. Enteral feeding was isolating, patients felt unhygienic^42^ and restricted in their physical activity.^43^ Xerostomia‐related dysphasia,^36^ the presence of a nasogastric tube^42^ or dysphagia^37^ resulted in social avoidance and consequent loss of opportunities for support^31^, ^46^:

I declined invitations… I did not want to ruin the appetite for everyone at the dinner table.^46^

Perceived stigma of an HPV+ve diagnosis also affected social identity.^33^ Likened to the *‘elephant in the room’* ^32^ patients withheld information: *‘…you don't go around broadcasting that something's sexually transmitted’,* ^33^ decreasing self‐esteem and affecting relationships:

When they told me it was because of HPV I don’t think I told him for ages … it was easier to tell other people why as opposed to him.’ ‘My partner (sic) …. “said something that made me feel really dirty.^33^

Its significance was reduced for some when its contagious nature and prevalence was understood.^33^ However, self‐identity within strained relationships was also challenged by changing roles^26^, ^43^:

…it sort of becomes a bit like a child mother relationship… I found it hard to move back to being an equal adult.^26^

Attempts to return to the role of family provider to alleviate financial concerns were thwarted by treatment consequences,^32^, ^42^, ^43^, ^44^ such as communication difficulties attributed with being perceived as less intelligent.^32^ These wide‐reaching changes affected a sense of self, exacerbated by an inability to undertake fundamental functions of living,^27^, ^29^ previously taken for granted or valued.^26^, ^32^, ^36^ The pleasure of eating was replaced with fear and dread, as it hurt to eat.^26^, ^36^, ^37^, ^47^ Being unable to enjoy the taste of culturally normative foods could also threaten identity.^46^, ^48^ Sleep deprivation and lack of energy had a significant influence on a person's sense of self^32^, ^36^:

It really does take your breath away and it takes your spirit away, which is even worse….^32^

The after‐treatment period was likened to *‘a black hole’* ^41^ and the accumulation of such disabling and distressing effects resulted in loss of interest and self‐worth:

I gave it up. I didn’t feel and I was not willing to do anything, I didn’t recognize myself anymore, this thing had left a shell of my previous self, a self that I despised.^36^

There was belief that the cancer was undeserved and unfair in a previously healthy person.^40^ Some did assume an illness identity,^42^, ^48^ which could be permanent,^43^ affecting future plans and life expectations.^28^, ^31^, ^32^ This view was reinforced by shifts in body image due to dental extraction,^42^ weight loss^37^, ^48^ and facial lymphoedema, also affecting sleep and ability to undertake swallowing exercises.^45^, ^49^ Self‐esteem was reduced and self‐consciousness raised, affecting social identity, particularly for female patients:

I try to cover it up when I go out… so that people don’t look at me.^45^

These clustered treatment consequences heightened their impact upon self‐identity.

### 3.6 Unrealistic expectations of recovery

This construct captured the emotional consequences of unmet expectations of recovery. Patients described the first weeks after treatment *‘….like a nightmare’*,^29^ including a long struggle,^31^, ^46^ feelings of failure^43^ and depression^31^, ^36^, ^42^:

You’re still mentally in a very bad place, and physically in a bad place… it took me a long time to come out of that bad place. I went through depression and all sorts of terrible things in that six months after treatment.^31^

The unexpected difficulty of recovering physically was amplified when considerable side effects during treatment initially worsened, rather than improved.^26^, ^27^, ^36^ Patients' hopes for a quick recovery were unfulfilled, creating uncertainty about the future^28^, ^29^, ^37^, ^41^, ^42^, ^43^, ^45^, ^46^, ^47^, ^50^:

…I didn’t expect it to be going on as long as it did… I thought that once the treatment had finished a couple of weeks and I’d be fine.^50^

Those expecting a long recovery were frustrated by a lack of progress, reporting difficulty sustaining motivation for rehabilitation.^45^ Discord was magnified by optimistic information provided regarding side effect likelihood and duration, such as mucous and saliva production and normal eating patterns^46^, ^47^, ^50^:

Most people tried to sort of sugar coat it a little bit and say ‘‘these things can happen but they do not always happen and they might not happen to you.”^50^

Although patients conceded they might not have absorbed all of the information given at clinic appointments^50^ their lived reality did not correspond with what they heard or read.^47^ This was echoed by those who sought to prepare, finding information had limited meaning in advance of the actual experience:

I could not imagine how it should be so I asked the doctor if there was something special to think about concerning the food, and the doctor said to me: that you eat. I could not understand how right that was, how difficult it should be.^46^

The fluctuating nature of side effects intensified uncertainty, raising doubt about improvement or permanent change, the meaning behind new symptoms and recovery time^27^, ^29^, ^37^, ^45^, ^49^:

What they say to you is you’re going to be very poorly and for a couple of weeks after, then things will start picking up. Well two weeks after, then a month after and you think well I’m still not eating, it’s on your mind, am I lagging behind people?^37^

Patients tried to assess the normality of their recovery but hearing *‘everyone's different’* ^31^ did not restore confidence. Concerns about the future included fears for the family, and existential thoughts of potentially not being there as a parent.^34^, ^42^ Anxieties about survival^40^ were intensified for those with unmet information needs regarding HPV:

… is it [cancer] more likely to come back because of this [HPV]? Is it something that stays in your body?^33^

Patients wanted to be better informed,^27^ proposing fellow patients could help.^41^ Some would want to know the worst that would happen,^50^ but others felt otherwise:

It was for the best that I did not know because then it would have felt impossible that I should be sick until June…. I thought all the time – next Friday it will be better.^46^

### 3.7 Finding ways to cope

The emotional rollercoaster patients described meant having to seek ways to cope, the fourth construct, often with assistance from others.^29^, ^41^, ^46^ Patients monitored for small signs of improvement, to bolster confidence.^46^ Self‐compassion was evident,^38^ although emotions were checked:

You have to be careful not to become a victim or have a victim mentality. You have to really work hard to say “Ok, I’ve got these things, I’ve to live with them now, let’s get on with it.” But that’s something you have to, almost on a daily basis; I find I’ve got to remind myself.^31^

Patients cultivated an optimistic, hopeful attitude^27^, ^28^, ^29^, ^30^, ^32^, ^41^, ^42^, ^43^, ^46^, ^47^ despite the uncertainty of recovery.^28^ Some were able to positively reframe difficulties:

I’m still the same bloke. The only little problem I’ve got, and I treat it as little, is my eating problem.^27^

Other coping strategies included goal‐setting^32^ (e.g., tube removal and eating orally^47^) and adopting a fighting spirit.^30^, ^38^, ^48^ Downward social comparison including positive self‐appraisal helped patients to situate themselves and compare themselves favourably.^28^, ^30^, ^43^, ^44^ Some found returning to work enabled resumption of some normality and aided coping, whilst others had to return to work before adequate recovery, due to financial obligations.^32^ Maladaptive coping strategies included disengagement through avoidance, fantasy thinking, self‐blame, or denial.^30^ Past coping strategies were not always available, such as eating,^48^ whilst social avoidance compromised support^31^, ^36^, ^46^:

I was afraid to talk to anyone, not from a close distance at least, I was afraid of their reaction to my bad breath…I didn’t know if I could handle this kind of rejection. It seemed better to be alone and silent and keep my dignity…perhaps the only thing that the treatment hadn’t take away from me, yet.^36^

However, social contact conveyed others cared for example, in the parental role^34^, ^42^ or through maintaining social traditions^32^, ^37^:

Went to the pub as before, despite having profound swallowing difficulties^37^

The support of fellow patients for sharing experiences was valued. Insightful peers enabled emotional expression, information provision, resolution of uncertainty and making sense of experiences^27^, ^31^, ^32^, ^41^, ^43^, ^50^:

When I met [another support group member] he said to me “it gets better”. And that was probably one of the best things that ever happened to me because at that stage I didn’t think it was ever going to get better.^31^

Informal caregivers, often partners, supported patients who notably described coping using the pronoun ‘we’.^26^, ^33^, ^41^ Accepting help was vital following treatment,^31^ as managing independently as before was impossible.^26^, ^27^, ^30^, ^41^, ^42^ Recognition of this was common^26^, ^27^:

My wife forced me to eat. She said: “Stop tube feeding. We are going to eat now.” .… My wife and children have pulled me through.^41^

However, some described a lack of understanding by family and friends reducing available support,^26^, ^27^, ^41^, ^48^ whilst caregiving was threatened by patients' fluctuating needs as side effect severity varied.^26^, ^27^, ^32^ Desire to reassert control during recovery could strain relationships, as patients' acceptance of practical help lessened:

Finally I said “Hey, back off . . . There are some things now that I want to be able to do.”^32^

Others reported shared experiences strengthened relationships,^32^ but awareness of the impact of the cancer diagnosis on the whole family and the need to comfort and support them, increased coping burden.^40^ Enteral feeding emerged as a significant feature within intimate relationships but could provide an opportunity to involve family in practical tasks.^43^

### 3.8 Adjusting to life after treatment

This construct identified how patients sought to adjust and regain control of their changed lives, for example, solving practical problems and adapting to physical changes^27^, ^31^, ^42^, ^43^, ^45^, ^48^, ^49^:

I can’t open my jaw wide enough, … when I eat a spoon full of something, I can’t. I have to put the spoon in sideways.^48^

Although acceptance of enteral feeding was perceived as losing control in an early study,^48^ more recently, increased empowerment through HCP‐led enteral feeding self‐management programs was expressed, enabling relief from worries about nutrition and weight loss.^42^, ^43^ Patients described opting for a Percutaneous Endoscopic Gastrostomy (‘G‐tube’) to live a normal life again, fostering a sense of control, and reducing social isolation as they re‐engaged with support networks.^43^ Understanding their own experience resulted in acceptance evolving^26^, ^27^, ^32^, ^42^, ^45^, ^49^:

I don’t really worry about it anymore . . . no point. If I can’t eat it I can’t eat it.^27^

However, for some, adjustment to what had been lost was harder^28^, ^36^, ^43^:

…he told me that it [G‐tube] was going to be permanent…I just can't imagine never eating again…^43^

The process of adjustment observed in some studies, particularly those with varied data collection time points included looking for signs of treatment success, or otherwise,^29^, ^42^ for example, changes in texture, swallowable portion sizes^37^ and less tangible signs, such as *‘getting better’*.^41^ Although individual time scales varied, this could help trigger a ‘Turning point’,^38^ which could be supported by others^32^, ^48^:

I said to [a friend] … “this is my new normal. . . do I necessarily like it? No, but it’s a whole lot better than the alternative”. He looked at me and said, “You know, that’s not a bad way to look at things.” And that’s the way I am.^32^

This ability to modify one's perspective, or psychological flexibility,^51^ was also demonstrated by those cautiously learning to live with uncertainty.^28^ A yearning to return to the activities of daily living was evident,^40^ whilst the significance of patients' families in the reassessment of priorities within the ‘new normal’ was clear^32^, ^34^, ^38^, ^40^:

Your values change. You value drinking and going out with you mates and working…. Then you step back from that and say, ‘Hold on’. Everybody thinks kids are important, but it makes them doubly important.^34^

And for some, ‘moving on’ could lead to benefits from ‘giving back’.^32^, ^43^

## 4 DISCUSSION

This review illustrated that after treatment HPV+ve oropharyngeal cancer patients experienced ‘gaps in continuity of support from HCPs’, ‘changes to self‐identity’ and ‘unrealistic expectations of recovery’, challenging them to ‘find ways to cope’, which if successful could enable them to begin ‘adjusting to life after treatment’. Figure 2 depicts these findings building upon past research,^8^, ^11^, ^12^ conveying ‘what it is like’ during early recovery for this population following (chemo)radiotherapy.

Awareness of gaps in continuity of support demonstrated unmet psychosocial needs. Opportunities to obtain timely information were limited by patients' reluctance to contact HCPs, likely due to stoicism. HCP‐led support for enteral feeding, reflecting UK practice development,^52^ did meet needs,^43^ indicating the benefits of a formalised approach. Although of secondary importance to cancer, HPV was a worry, which if unaddressed, persisted into survivorship.^33^ Recognition of this by the European HNC Society's ‘Make Sense Campaign’ has resulted in HCP patient support guidance,^53^ acknowledged as required in the UK.^9^, ^54^

Middle adulthood^55^ is a life stage when expectations and goals (e.g., raising a family and work plans) are often formulated and realised. This working age population experienced considerable interlinked shifts in their self‐identity and life assumptions, termed ‘biographical disruption’,^56^ previously recognised within HNC.^8^ There was a contrast between the challenges of having to depend on others and severe side effects, after limited pre‐treatment symptoms and news of an optimistic prognosis. Disappointment when hopes for a swift recovery were not met, exacerbated uncertainty about the future, following an unexpected cancer diagnosis. HPV+ve status, visible signs of illness, such as a nasogastric tube, loss of control and the sudden switch in role from full time employment to full time self‐management, also threatened identity. Subsequent social isolation could be amplified by withholding an HPV+ve diagnosis,^33^ threatening supportive relationships, and potentially causing further disruption.

Although patients may have received information, they had unrealistic expectations of recovery within their own personal, family, work, and social context, and a discordance between being told what may happen and lived reality persisted. Assessment by patients of uncertainties as ‘dangerous’,^57^ such as HPV‐associated recurrence risk, fluctuating side effects or unknown permanence of changes, challenged their management, impacting coping. Uncertainty has been linked to lower quality of life in HNC^58^ and prostate cancer.^59^ Additionally, focus upon physical effects after (chemo)radiotherapy, during ‘Survive mode’^38^ may mask emotional support needs, previously found to be greater in younger HNC patients than older.^60^ Depression was disclosed by patients within this review^31^, ^36^, ^42^ and previously in up to one third of HNC patients following radiotherapy.^61^ High levels of distress have been associated with avoidant coping strategies such as distancing and disengagement^62^ and reduced quality of life,^63^ pertinent for this population requiring motivation to undertake self‐management, such as swallowing exercises.^64^ Support includes re‐habilitation programmes, stress management, relaxation therapy^53^ and person‐centred interventions.^65^ Counselling and Cognitive Behavioural Therapy (CBT) have also been widely used to treat depression in cancer patients,^66^ but require further research within HNC.^67^

Self‐appraisal, as patients sought ways to cope, was evident throughout these studies for example, questioning if experiences were normal, reluctance to contact HCPs *‘too soon’* and goal monitoring. Past experiences and personality disposition types affected individual coping styles and patients' information needs. Without personalised assessment (e.g., ‘Patient Concerns Inventory’ (PCI)^68^ and Macmillan Cancer Support Holistic Needs Assessment (HNA)^69^) these may be unmet, causing greater anxiety than that related to side effect severity.^70^

Finding ways to cope enabled responses to the biographical disruption experienced, determined by different cognitive models of self, world, and others, including role flexibility, priority reassessment and social comparison. This adaptation of their ‘Assumptive world’^71^ made a positive transition possible, if needs such as HPV knowledge and returning to work challenges were met. Such potential barriers to ‘finding a path’^8^ following treatment were portrayed here, and previously,^12^ as a ‘sense of abandonment’. Subsequent searching for meaning, whilst facing loss of control and ongoing uncertainty, have previously been described^8^ following a past exploration of ‘liminality in illness’.^72^ Interviewed colorectal cancer patients were able to avoid the sense of alienation, or ‘separateness’ conceptualised if supported by someone who had undergone similar experiences. That *‘traumatic experiences are indescribable until they have been experienced’* has been demonstrated within HNC,^70^ where expectations during recovery were revised. Peer support has enhanced coping during HNC radiotherapy,^73^ providing peers were well‐matched, and was echoed here, after treatment. Patients were able to attribute personal meaning to credible information from peers, reducing uncertainty. Sharing experiences in support groups, where social confidence could be regained, or during chance conversations in clinic established a sense of commonality, normalising experiences and creating precedence: a ‘frame of reference’^74^. Within the development of their ‘Information seeking behaviour theory’, these researchers found that information from HCPs *‘took a backseat’* to the importance placed on patients' lived experience. It seems that through shared social reality, peers are able to convey information in a unique way, bridging gaps in support.

The ability to modify one's relationship with experiences, or psychological flexibility, may also help meet the psychosocial needs of this population, some of whom were able to reframe experiences positively (e.g., ‘a new normal’ and strengthened relationships). Additionally, parents of young children and those returning to work provided examples of a drive to adjust to a changed self, a ‘Turning Point’,^38^ reflecting Leventhal's ‘Self‐Regulatory Model’.^75^ Acceptance and Commitment Therapy (ACT),^51^ a supportive intervention to enhance psychological flexibility, may help facilitate adjustment to the complex psychosocial experiences identified here.

### 4.1 Study limitations

This meta‐ethnography interpreted the findings of studies posing varied research questions to reveal psychosocial experiences and support needs of HPV+ve oropharyngeal cancer patients following (chemo)radiotherapy. Any preconceptions in interpretations by the first author, a therapeutic radiographer, were addressed by confirming findings with the co‐authors. As qualitative research includes potential biases, for example, study participant self‐selection and recall of experiences, findings were not generalisable to all HNC patients. Trustworthiness, including credibility and dependability, were demonstrated through rich description and confirmation of constructs, for example, psychosocial impact conveyed when each paper or topic occurred within every construct, such as experience related to food and eating. However, synthesis of papers with varied research approaches or with specific foci (evident in more recent studies e.g., HPV diagnosis, enteral feeding, lymphoedema) meant not all papers demonstrated every construct, thus limiting their influence (e.g., some included few quotations,^29^, ^38^, ^44^ whilst deductive approaches were less likely to reflect every construct^31^, ^41^).

Study characteristics (Table S3) were used to monitor and limit influence, for example, older studies including surgery only patients^34^, ^44^ or untypically large female populations.^36^, ^47^ Populations were mainly Caucasian and male with an average age below 65 years, reflecting past HPV+ve oropharyngeal cancer incidence, however, experiences may not represent those of the growing number of female patients.^2^ Practice development differences such as pre‐habilitation and HCP role in fostering self‐management over time and between countries, created inconsistencies, affecting the strength of some constructs.

### 4.2 Clinical implications

The UK HNC MDT guidelines^76^ were introduced during the timespan of these studies, enhancing support. Personalised care^77^ has been facilitated by tools (e.g., PCI,^68^ HNA^69^) to ascertain unmet needs and engage patients in self‐management. Additionally, this review found that HPV+ve oropharyngeal cancer patients require:

- Specific support to cope with severe side effects, changes in self‐identity and treatment impact (e.g., disruption, sense of uncertainty) to promote and sustain self‐management and adjustment.
- Clear information guiding expectations around recovery timelines, side effect profile and return to work implications to mitigate gaps in support and work towards alignment of expectations and experiences.
- Access to tailored HPV‐related information, involving HCP education.^54^
- Support for patients as parents, for relationships and families.
- Facilitation of well‐matched peer support for example, support groups, buddy schemes.
- Enhanced psychological support for example, CBT, ACT.

## 5 CONCLUSIONS

This meta‐ethnography has highlighted the considerable psychosocial needs of HPV+ve oropharyngeal cancer patients during early recovery following (chemo)radiotherapy. It builds upon past HNC reviews by revealing the experience of this population within five interlinked constructs, conveying the complexity of experience, and describing ‘what it is like’. Despite often receiving support from others and a favourable prognosis, an emotional rollercoaster meant patients struggled to reconcile their experiences with expectations of recovery. Gaps in timely, tailored information and available support challenged their ability to cope after treatment. Poor psychosocial well‐being, in what may be long cancer survivorship could threaten an otherwise successful outcome, which may require enhanced provision. Evolving understanding of HPV+ve oropharyngeal cancer patients' experiences following (chemo)radiotherapy should enable facilitated adjustment through personalised support and tailored interventions.

## CONFLICT OF INTEREST

The authors have no conflict of interest to declare.

## ETHICS APPROVAL

Ethical approval was not required as this was a review.

## Supporting information

Table S1

Table S2

Table S3

## Data Availability

The data that support the findings of this review are available from the corresponding author upon reasonable request.
