# Supplementary material for: The psychosocial experiences of human papillomavirus (HPV) positive oropharyngeal cancer patients following (chemo)radiotherapy: A systematic review and meta‐ethnography
Source: Psychooncology. 2022 Jul 5;31(12):2009–19. doi: 10.1002/pon.5984 (PMC10084069; doi:10.1002/pon.5984)
Supplement: Supplementary file 1 — Table S1 [file PON-31-2009-s002.pdf]

Supplementary Information 1: CINAHL search

Search 1
TI ((head AND neck) OR "upper aerodigestive tract" OR oropharyn* OR oro-pharyn* OR tonsil OR oral OR hypopharyn* OR laryn* OR mouth) OR AB ((head AND neck) OR "upper aerodigestive tract" OR oropharyn* OR oro-pharyn* OR tonsil OR oral OR hypopharyn* OR laryn* OR mouth)

Search 2
TI (cancer* OR neoplasm* OR tumor* OR tumour* OR carcinoma*) OR AB (cancer* OR neoplasm* OR tumor* OR tumour* OR carcinoma*)

Search 3
S1 AND S2

Search 4
(MH "Head and Neck Neoplasms") OR (MH "Squamous Cell Carcinoma of Head and Neck") OR (MH "Tonsillar Neoplasms") OR (MH "Oropharyngeal Neoplasms") OR (MH "Hypopharyngeal Neoplasms") OR (MH "Pharyngeal Neoplasms") OR (MH "Laryngeal Neoplasms") OR (MH "Tongue Neoplasms") OR (MH "Palatal Neoplasms") OR (MH "Mouth Neoplasms")

Search 5
S3 OR S4

Search 6
TI (after* OR follow* OR surviv* OR post OR post-treatment OR posttreatment OR “end of treatment” OR “completion of treatment” OR “living with”) OR AB (after* OR follow* OR surviv* OR post OR post-treatment OR posttreatment OR “end of treatment” OR “completion of treatment” OR “living with”)

Search 7
(MH "Survivorship")

Search 8
S6 OR S7

Search 9
TI (chemoradiotherapy OR chemoradiation OR radiotherapy OR radiation) OR AB (chemoradiotherapy OR chemoradiation OR radiotherapy OR radiation)

Search 10
(MH "Chemoradiotherapy") OR (MH "Chemoradiotherapy, Adjuvant") OR (MH "Radiotherapy")

Search 11
S9 OR S10

Search 12
TI (patient*) OR AB (patient*)

Search 13
(MH "Cancer Patients") OR (MH "Patients") OR (MH "Cancer Survivors")

Search 14

S12 OR S13

Search 15
TI (psychosocial* OR psycholog* OR social* OR emotion* OR need* OR concern* OR “quality of life” OR “quality-of-life” OR depress* OR anxiet* OR information* OR communication OR spiritual OR support* OR well-being* OR wellbeing OR distress* OR stress OR “fear of recurrence” OR “fear of cancer recurrence” OR “fear of progression” OR experienc* OR coping OR cope OR self-efficacy OR self-esteem OR adjust* OR adapt* OR accept* OR self-blame OR isolat* OR stigma OR body image OR sexual* OR intima* OR helpless* OR hope* OR avoid* OR worry OR guilt OR “supportive care needs” OR uncertainty) OR AB (psychosocial* OR psycholog* OR social* OR emotion* OR need* OR concern* OR “quality of life” OR “quality-of-life” OR depress* OR anxiet* OR information* OR communication OR spiritual OR support* OR well-being* OR wellbeing OR distress* OR stress OR “fear of recurrence” OR “fear of cancer recurrence” OR “fear of progression” OR experienc* OR coping OR cope OR self-efficacy OR self-esteem OR adjust* OR adapt* OR accept* OR self-blame OR isolat* OR stigma OR body image OR sexual* OR intima* OR helpless* OR hope* OR avoid* OR worry OR guilt OR “supportive care needs” OR uncertainty)

Search 16
(MH "Support, Psychosocial") OR (MH "Psychosocial Aspects of Illness") OR (MH "Rehabilitation, Psychosocial") OR (MH "Stress, Psychological") OR (MH "Depression") OR (MH "Anxiety Disorders") OR (MH "Generalized Anxiety Disorder") OR (MH "Social Anxiety Disorders") OR (MH "Stress Disorders, Post-Traumatic") OR (MH "Anticipatory Anxiety") OR (MH "Psychosexual Disorders") OR (MH "Intimacy")

Search 17
S15 OR S16

Search 18
S5 AND S8 AND S11 AND S14 AND S17
