# Supplementary material for: The psychosocial experiences of human papillomavirus (HPV) positive oropharyngeal cancer patients following (chemo)radiotherapy: A systematic review and meta‐ethnography
Source: Psychooncology. 2022 Jul 5;31(12):2009–19. doi: 10.1002/pon.5984 (PMC10084069; doi:10.1002/pon.5984)
Supplement: Supplementary file 2 — Table S2 [file PON-31-2009-s001.pdf]

Supplementary Information 2: Example of Synthesis

Paper	Author's 2nd order constructs	Patient quotes – 1st order constructs	3rd order constructs of new synthesis				
			Gaps in continuity of HCP support	Changes to self-identity	Unrealistic expectations of recovery	Finding ways to cope	Adjusting to life after EOT
Peeters et al<br><br>(2018) Nether-lands<br><br>'Support needs of people with HNC regarding the disease and its treatment'	Instrumental support	'That there is a coordinator who will keep an eye on things . . . that the follow-up from the nurse practitioner is continued, also for information about referral to relevant care professionals. Because you really don't have an idea [about the possibilities].'<br>'I knew I could contact [the hospital] for support. . . . As the nurse practitioner says: "If something is wrong, just give a call." But you don't want to do that too soon. Still a kind of threshold, I think. You first wait for better times, just one more week.'<br>'My wife forced me to eat. She said: "Stop tube feeding. We are going to eat now." . . . And then she started with small appetizers, a piece of fish, a piece of . . . everything that was smooth. Because I had difficulty swallowing. . . . My wife and children have pulled me through. I did not want it at the time.'	Continuity of care and tailored support needed<br><br>Uncertain of when to seek help		Waiting to get better	Caregiver's support	
	Psychosocial support	"[I felt] somewhat abandoned. For support after treatment, it would be better to have follow-up continued. . . . This could be part of the tasks of a coordinating nurse." 'a black hole'	Insufficient support after treatment				

		‘Previously, I was always the first to finish dinner. . . . Nowadays, however, I am the last to finish eating and [at home] they are sometimes joking about that. . . . We all need a little bit of self-mockery; I think it is good to see things positively.’<br>“That you can share things with each other, about experiences and feelings, and about how to do things. Then you can maybe relativize the severity of your own situation.”				Use of humour for positive outlook<br><br>Peer support (lateral social comparison)	
	Relational support	“I felt that I was getting better, so I stopped my tube feeding and started to eat some food. I just tried it and it went quite well. . . . [I thought]: If it doesn’t work, it doesn’t work.”<br>‘Of course, you have to do it on your own. . . . But in these kinds of situations, you do not always want [HCPs] to ask you what you prefer. They should just say, “We are going to do it this way.”<br>“Honestly, it seemed as if [my family] did not exactly realize what was happening.”<br>‘People close to you come to visit you for about one hour, but from the moment they leave, they go straight back to their own lives and do not have any idea of the emotional and physical impact of the disease.’<br>“I was not at all prepared for the fact that you can come out of the treatment in such a bad state. [Fellow patients] could have been helpful there.”	Tailored support for needs wanted e.g., timely lead from HCPs		Expectations not met	Monitoring - problem focused coping<br><br>Needing to feel understood by those close<br><br>Value of shared experience	Understanding self
