# Supplementary material for: The psychosocial experiences of human papillomavirus (HPV) positive oropharyngeal cancer patients following (chemo)radiotherapy: A systematic review and meta‐ethnography
Source: Psychooncology. 2022 Jul 5;31(12):2009–19. doi: 10.1002/pon.5984 (PMC10084069; doi:10.1002/pon.5984)
Supplement: Supplementary file 3 — Table S3 [file PON-31-2009-s003.pdf]

Supplementary Information 3: Data Extraction table showing characteristics of the 23 selected papers

First author, year, country	Study Title	Study design	Sample: total, % Oropharyngeal patients MF, age	Treatment	Sampling method and time point of data collection	Method of data collection	Method of data analysis
1. Semple et al (2010)<br><br>NI, UK	Experience of parents with HNC who are caring for young children	Exploratory descriptive study	12 HNC, unspecified site,<br>10 M, all <59 yrs.	9 had radiotherapy, 4 + chemotherapy 5 + surgery, 3 had surgery only.	Purposive: diagnosis within 3 yrs. when had children aged 16 or under	Interviews with topic guide & open-ended questions	Cognitive mapping – modelling technique to show ideas, beliefs, values, and attitudes & their relationships to each other. 'How the world is seen'.
2. Andersen and Jarden (2012) Denmark	Coping with RT for HNC: a qualitative exploration	Hermeneutic approach to interpretative study	5 HNC, includes oropharyngeal patient(s),<br>4 M, 50-65 yrs.	All had radiotherapy	Convenience: within one month of treatment	Semi-structured interviews with topic guide	Thematic analysis with application of Lazarus & Folkman conceptual model of coping
3. Haisfield-Wolfe et al (2012)<br><br>USA	Perspectives on coping among HNC patients receiving radiotherapy	Longitudinal descriptive study	21 HNC, 16 oropharyngeal patients (76%) 18 M,<br>av. 59.2 yrs.	All had radiotherapy, 15 had concomitant chemotherapy.	Convenience.<br>4 time points: Radiotherapy week 1, 5, 9 & 12	Interviews with 3 open-ended questions	Content analysis
4. Molassiotis and Rogers (2012)<br><br>UK	Symptom experience and regaining normality in the first year following a diagnosis of HNC	Longitudinal interpretative explanation	16 HNC, 14 oral and oropharyngeal 14 M,<br>av. 61 yrs. 34-80	12 had radiotherapy, 4 had concomitant chemotherapy, 4 had surgery only.<br>Half palliative	Purposive sampling for variation:<br>4 time points: diagnosis, 3, 6 (EOT) & 12 months	Interviews: questions used for varied cancer sites & early responses informed questions at later interviews	Content analysis cross-sectionally and longitudinally
5. Moore et al (2014)<br><br>Australia	'I have quality of life but…' Exploring support needs important to quality of life in HNC	Exploratory descriptive study of experiences throughout treatment trajectory	8 HNC, 3 oropharyngeal = 38%.<br>7 M, 51-60 yrs. av. 60	All had radiotherapy, 5 also had surgery.	Snowball and convenience sampling from a support group.<br>1-8yrs after treatment, av.= 4.25yrs.	Semi-structured interviews with open-ended questions	Content analysis – inductive and directed using Lazarus & Folkman model of stress.

6. Schaller et al<br>(2015)<br><br>Sweden	Experiences of pain: a longitudinal study of HNC patients recently treated with radiotherapy	Longitudinal descriptive study	26 HNC, 9 (35%) oropharyngeal<br>19 M, av. 64 yrs.	All had radiotherapy, 11 + chemotherapy + varied surgery	Purposive sampling.<br>1 and 4-6 months after treatment	2 interviews – unstructured	Content analysis
7. McQuestion and Fitch<br>(2016)<br><br>Canada	Patients' experiences of receiving RT for HNC: before, during & after treatment	Interpretative descriptive study	17 HNC<br>(mixed site not specified),<br>12 M, 15 <70 yrs.	All had radiotherapy, none had chemotherapy	Purposive sampling - subset of larger study.<br>3-4 months after radiotherapy	Interviews plus member checking with focus group (4)	Thematic analysis (Giorgi '85)
8. Peeters et al<br>(2018)<br><br>Netherlands	Support needs of people with HNC regarding the disease and its treatment	Phenomeno-logical study using conceptual framework	13 HNC, 7 (54%) oropharyngeal<br>10 M, 48-73 years (median = 60)	All had radiotherapy, 9+ chemotherapy	Purposive sampling: 7 for 2 focus groups & 6 interviews.<br>6 <12months after RT, 7<2yrs	2 Focus groups and 6 interviews	Direct content analysis using Dwarswaard et al '16 conceptual framework of Self-management support needs.
9. Grattan et al<br>(2018)<br><br>Canada	Experiences of HNC in middle adulthood – consequences and coping	Interpretative descriptive study	10 middle aged HNC: 5 HPV+ve (& 4 untested)<br>9 M, <65yrs, median = 54	All had radiotherapy, 8 with surgery.	Purposive sampling.<br>2-19 months after treatment, median = 14	Unstructured interviews	Thematic analysis
10. Dodd et al<br>(2019)<br><br>UK	Psychosocial impact of HPV HNC diagnosis on patients and their partners: a qualitative interview study	Descriptive study	20 HPV+ oropharyngeal patients (14 M) and 12 partners (2 M) median age = 59, 41-83.	18 had radio-therapy, 5 + chemotherapy<br>1 had surgery only	Purposive sampling. 1 year after diagnosis, range = 12-53 months, av.= 23	Semi-structured interviews	Thematic analysis – focused on the 12 patients & 5 partners aware of HPV+ve status
11. Calver et al<br>(2019)<br><br>UK	How patients adjust psycho-logically to the experience of HNC: a grounded theory	Grounded theory	12 HNC, 4 (33.3%) oropharyngeal inc.1 rec.<br>8 M, 35-72 yrs. Av 58.75	8 had radiotherapy, 4+ chemotherapy.<br>1 at diagnosis, 3 surgery only.	Purposive and then theoretical sampling. Only 2 oropharyngeal patients treated within 2 years.	Semi-structured interviews – questions refined through study.	Analysis guided by Corbin & Strauss '15.

12. McQuestion et al (2011)<br><br>Canada	Changed meaning of food – physical emotional social loss for patients having received HNC radiation treatment	Interpretative descriptive study	17 HNC (mixed site not specified),<br>12 M, 15 <70 yrs.	All had radiotherapy, none had chemotherapy.	Purposive sampling - subset of larger study.<br>3-4 months after radiotherapy	Interviews	Thematic analysis (Giorgi '85)
13. Ottosson et al (2013)<br><br>Sweden	The experience of food, eating and meals after RT for HNC	Descriptive study	13 HNC, 6 (46%) oropharyngeal 11 M, median 60 yrs. 47-70	All had radiotherapy, none had chemotherapy.	Purposive sampling. 8-11 months after RT - with reflection on early post RT	Semi-structured interviews	Content analysis
14a. Nund et al (2014a)<br><br>Australia	Survivors' experiences of dysphagia-related services following HNC: Implications for clinical practice	Descriptive with phenomeno-logical aspects	24 HNC, 18 (75%) oropharyngeal (14 HPV+ve), 20 M, 18 <65 yrs.	23 had chemo-radiotherapy, 1 had radiotherapy alone.	Purposive sampling: have dysphagia. Av = 18 months after treatment -range 1-60mo, 5< 3mo	Semi-structured interviews	Thematic analysis
14b. Nund et al (2014b)<br>Australia	Lived experience of dysphagia following non-surgical treatment for HNC	As above	As above	As above	As above	As above	As above
15. Charalambous (2014)<br><br>Cyprus	Hermeneutic phenomeno-logical interpretations of HNC patients experiences living with radiation-induced xerostomia: the price to pay?	Hermeneutic phenomeno-logy	15 HNC, (site not specified) 8 M, 39-66 yrs. (av. 54)	All had radiotherapy	Purposive sampling: including assessment of xerostomia.<br>3 months after treatment	Unstructured interviews	Thematic analysis and hermeneutic phenomenological structural analysis

16. Brockbank et al (2015)<br><br>UK	Pre-treatment Information on Dysphagia: Exploring the views of HNC patients	Descriptive study of service experience	24 HNC (site not given)<br>22 M, median 59 yrs. (44-77)	All had chemo-radiotherapy	Purposive sampling: had dysphagia. Av. 10 months, 2-22 months after treatment	Focus groups (11) and interviews (13)	Thematic analysis
17. Patterson et al (2015)<br><br>UK	HNC patients' perceptions of swallowing following chemo-radiotherapy	2 stages: Ethnographic observation & descriptive study	12 + 9 HNC, 39% oropharyngeal *(approx. from larger study).*<br>18 M, age 45-77 median 63.	All had radiotherapy, 17 also had chemotherapy.	Purposive sampling from larger study: presence of dysphagia.<br>3-18 months after treatment	Observations and interviews	Thematic analysis
18. Ehrsson et al (2015)<br><br>Sweden	HNC perceptions of QOL and how it is affected by the disease and enteral tube feeding during treatment.	Longitudinal descriptive study	36 HNC, 16 (39%) oropharyngeal<br>28 M, av. 62.3 yrs.	All had radiotherapy, 13 + chemotherapy, 12 + surgery	Purposive sampling. Pre-radiotherapy and 2 weeks & 3 months after treatment	Structured interviews, using QoL questionnaire.	Content analysis
19. Williams et al (2019)<br><br>UK	Patients' experience of enteral feeding following chemo-radiotherapy for HNC: a qualitative study	Interpretative descriptive study	10 HNC**,** 8 (80%) oropharyngeal<br>8 M, median 54, yrs. 43-65	All had radiotherapy, 9 + chemotherapy	Purposive sampling: presence of feeding tubes. Median 26 days after treatment, range 3-231 days, 9 patients within 12 weeks	Semi structured interviews	Thematic analysis
20. Sandmael et al (2019)<br><br>Norway	Nutritional experiences in HNC	Descriptive study	10 HNC, 7 (70%) oropharyngeal<br>5 M, median 59 yrs. 49 – 70	All had radiotherapy	Purposive sampling: from a supportive nutrition rehab intervention pilot study.<br>2-4 weeks after treatment	Semi structured interviews	Content analysis

21. McGarvey et al (2014)<br><br>Australia	Lymphoedema following treatment for HNC: impact on patients, and beliefs of HCPs	Interpretative descriptive study of patients and HCP beliefs	10 HNC, 5 oropharyngeal 8 M, av. 60.1 yrs. 32–75 and 10 HCPs.	All had radiotherapy, 5 non-oropharyngeal + neck dissection	Purposive sampling: presence of lymphoedema. Most around 12 months after treatment, all <18 months.	Interviews with open and closed questions	Thematic analysis
22. Jeans et al (2018)<br><br>Australia	Patient perceptions of living with head and neck lymphoedema and the impacts to swallowing, voice and speech function.	Interpretative descriptive study	12 HNC, 7 (58%) oropharyngeal (6 HPV+ve), 8 M, av. 61.3 yrs. 52–72	All had chemo-radiotherapy, 6 + neck dissection	Purposive sampling: presence of lymphoedema. 3-22 months (av. 8.4) after treatment	Semi structured interviews. Patients had questions beforehand, mixed face to face and phone	Thematic analysis
